# Fatal Hemoptysis With Pleural Effusions Secondary to Superior Vena Cava Obstruction as a Complication of Central Venous Catheterization

**DOI:** 10.7759/cureus.59690

**Published:** 2024-05-05

**Authors:** Shaheryar Usman, Muhammad Azaz I Cheema, Saleem Mustafa, Asma Iftikhar

**Affiliations:** 1 Internal Medicine, Northwell Health, Port Jefferson, USA; 2 Pulmonary and Critical Care Medicine, Northwell Health, Port Jefferson, USA

**Keywords:** severe anemia, bloodless surgery, jehovah's witness, hemoptysis, hemodialysis catheter, superior vena cava syndrome

## Abstract

This report outlines a rare case of superior vena cava (SVC) syndrome presenting with hemoptysis in a 33-year-old female Jehovah's Witness patient with a complex medical history, including systemic lupus erythematosus (SLE) and chronic hemodialysis dependency due to end-stage renal disease and a failed renal transplant. The SVC syndrome was attributed to occlusion from a right subclavian dialysis catheter. The management of this case was particularly challenging due to the patient's severe anemia and the development of a tension hemothorax following thoracentesis, compounded by her refusal of blood transfusions in adherence to her religious beliefs. A multidisciplinary approach, incorporating bloodless medical techniques such as erythropoietin and iron infusions alongside surgical interventions without blood transfusion, was successfully employed. This case sheds light on the evolving etiology of SVC syndrome and highlights the uncommon but potentially fatal occurrence of hemoptysis as a complication. It also emphasizes the importance of respecting patient values in complex medical decisions.

## Introduction

The first known description of superior vena cava (SVC) obstruction was provided by William Hunter in 1757 [1]. SVC syndrome is a condition where blood flows through the SVC, and its branches are partially or completely obstructed, leading to symptoms such as facial swelling, shortness of breath, and coughing [2]. In rare cases, hemoptysis may also manifest as an initial symptom in cases of SVC syndrome [2]. While this condition was traditionally associated with mediastinal malignancies, its etiology has seen a shift due to the increased use of dialysis catheters [3]. This case report describes a unique presentation of SVC syndrome in a Jehovah's Witness patient who presented with hemoptysis and was later found to have SVC syndrome attributed to her dialysis catheter. A significant challenge was encountered when severe anemia with a hemoglobin level of 2-3 g/dl developed, and the patient's family, respecting her wishes, refused blood transfusions. This refusal posed a dilemma in managing her condition, especially after she developed a tension hemothorax following a thoracentesis for pleural effusion, a complication of her SVC syndrome.

## Case presentation

**Figure 1:**
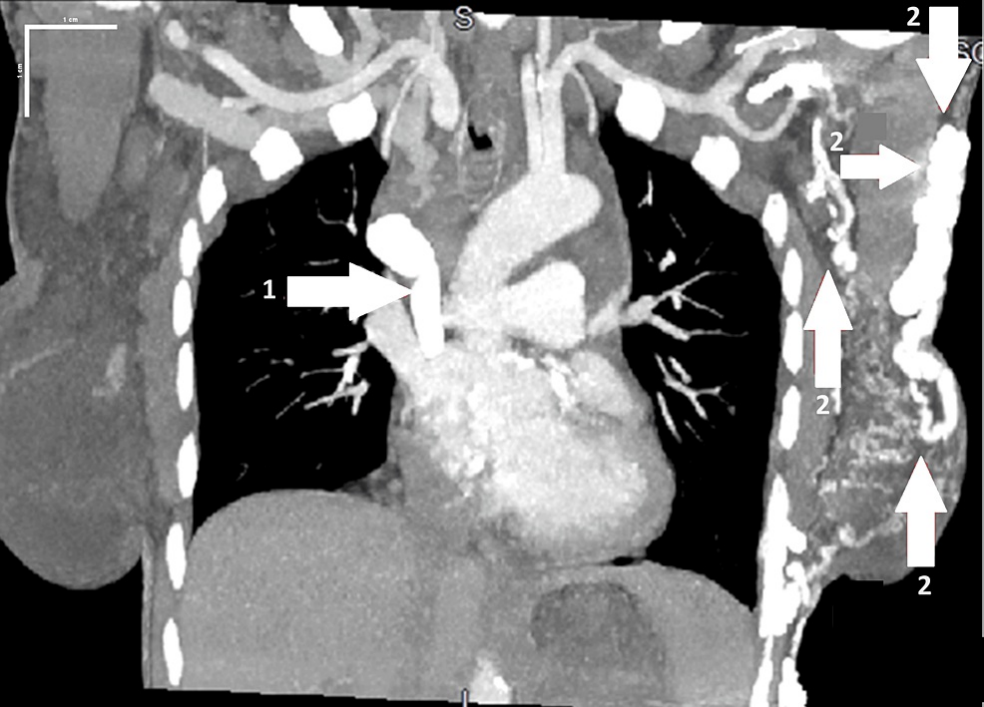
Coronal section computed tomography angiography (scale bar, 1 cm) showing via label 1: a hypertrophied azygos vein and via label 2: hypertrophied venous collateral vessels along the left chest wall.

**Figure 2:**
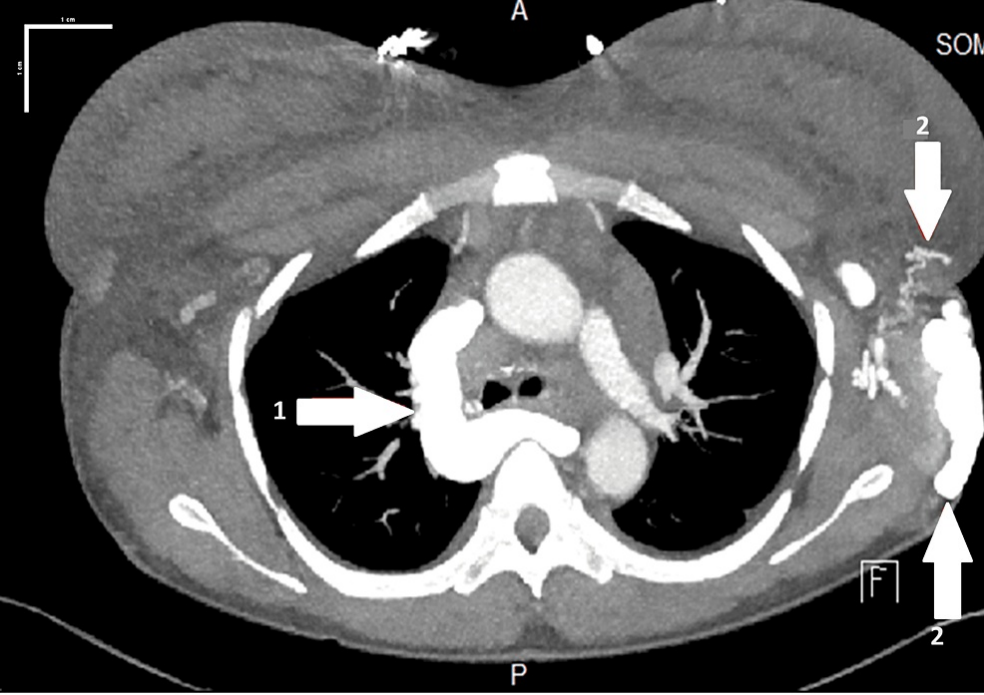
Axial section computed tomography angiography (scale bar, 1 cm) shows, via label 1: a hypertrophied azygos vein, and via label 2: hypertrophied venous collateral vessels along the left chest wall.

A 33-year-old female Jehovah's Witness with a history of systemic lupus erythematosus (SLE), complicated by lupus nephritis, failed renal transplant, and end-stage renal disease on dialysis, came to the hospital with a complaint of hemoptysis for three days. She was on dialysis thrice weekly through a right-side dialysis catheter. Computed tomography angiography (CTA) showed widespread hypertrophy of venous collateral vessels in the left chest wall and a significantly enlarged azygos vein. Additionally, edema within the chest, predominantly on the left side, may be attributed to obstruction of the patient's SVC (Figures 1, 2).

**Figure 3:**
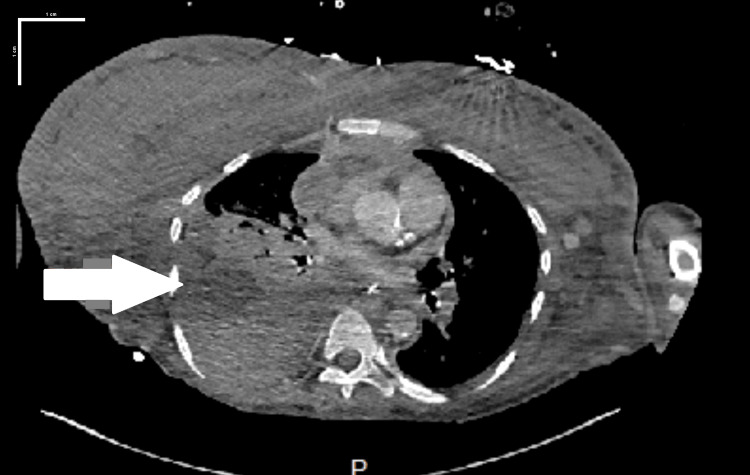
Axial section computed tomography angiography (scale bar, 1 cm) showing left-sided hemothorax.

The hemodialysis (HD) catheter was removed, and a tunnel left femoral HD catheter was placed for dialysis. The onset of SVC syndrome was unclear, but collateral maturity indicated that it was chronic. The patient went for SVC recanalization but failed treatment. In the meantime, an AV fistula was made in the left forearm. Hemoptysis was attributed to the extensive network of collateral vessels identified on the CT scan. A venogram, guided by interventional radiology (IR) of both upper extremities, revealed well-developed collateral networks and complete occlusion of the subclavian vein. This condition likely led to congestion in the pulmonary vessels, triggering hemoptysis. Her hemoglobin (Hb) levels were stable, and no active bleeding was noted on the venogram. Initial treatment with tranexamic acid to manage the hemoptysis proved ineffective. Subsequently, the patient was intubated for a bronchoscopy, which identified the source of bleeding to the right middle and lower lobes. She then successfully underwent embolization of the right bronchial artery by IR. Following this procedure, she experienced no further hemoptysis and was discharged. During a later visit, the patient returned to the emergency department complaining of shortness of breath. A chest X-ray showed that the right pleural effusion had grown in size compared to previous visits. As a result, the patient underwent a CT-guided thoracentesis. Unfortunately, during the procedure, she developed respiratory distress, which led to the development of a large right-sided tension hemothorax. She was moved to the ICU for further management, where she experienced an episode of cardiac arrest but was successfully resuscitated after 10 minutes. Her condition required multiple vasopressors due to shock. Her Hgb, initially 12.2 mg/dl at the time of admission, down-trended to 6.5 mg/dl. A repeat chest CT scan showed a substantial right hemothorax (Figure 3), displacing the heart and mediastinal structures to the left, indicative of tension physiology. The scan did not reveal any active bleeding.

The patient declined blood products and, after extensive consultation, was treated with erythropoietin, iron infusions, and folic acid. She underwent right-side video-assisted thoracoscopic surgery (VATS) to remove a retained hematoma and partially decorticate the right lung. After the procedure, her hemoglobin levels improved over a couple of weeks to 8.6 mg/dl, and she was weaned off pressors and extubated. Once the patient's clinical stability was achieved, the cardiothoracic surgeon discussed the next steps with the patient and family. The available options were either to instill tissue plasminogen activator (tPA) in the right chest cavity to liquefy the clot, which could result in catastrophic bleeding, or to take the patient back to the operation theater for more decortication, which could also lead to catastrophic bleeding due to the high-pressure venous system resulting from the SVC syndrome. However, the patient continued to decline blood products and was instead treated with erythropoietin (10,000 IU/0.5 mL) every second day, along with intravenous iron and B12 supplements. The patient was also found to have Staphylococcus epidermidis bacteremia, for which she received IV vancomycin as per infectious disease recommendations. Unfortunately, the patient suffered another cardiac arrest during their hospital stay, and the family decided to stop resuscitation and transition to comfort care.

## Discussion

Our case highlights the rare occurrence of hemoptysis as a presentation of SVC in a Jenoah Witness patient with severe anemia. Our case also explores the pathophysiology and management options for hemoptysis in the setting of dialysis catheter-associated SVC.

Previously, SVC was chiefly linked to mediastinal malignancies. However, its etiology has evolved over the years with the increased use of hemodialysis catheters [3]. Labriola et al. reported SVC stenosis in 9.4% of the cases (0.14/1000 catheter days) in chronic hemodialysis patients with tunnel-cuffed catheters [4]. Echefu et al. reported an increasing incidence of central venous obstruction with an increasing number of central venous catheters used, with a higher incidence noted in patients with a history of subclavian vein catheterization [5]. These studies accentuate the significance of selecting and monitoring vascular access in patients on long-term hemodialysis.

The pathophysiology of SVC syndrome involves endothelial damage and thrombosis, exacerbated in patients with hypercoagulable states like SLE [6]. This was evident in our case, where chronic venous cannulation likely contributed to SVC thrombosis. Patients with SVC syndrome typically present with head, neck, and arm edema, cyanosis, and distended collateral vessels. However, the severity and presentation vary based on the degree of SVC narrowing and the speed of onset [7]. 

Hemoptysis in SVC syndrome is an exceedingly rare presentation, with only a handful of cases (Table 1) reported between 2010 and 2023, as mentioned in Table 1. The exact mechanism of the hemoptysis has not been described previously; however, Tannu and colleagues proposed a similar mechanism in patients with mitral stenosis, where they documented bronchial varices. In mitral stenosis, the back pressure and the increased left atrial pressure can lead to bronchial varices that can easily bleed [8]. In our case, bronchoscopy revealed active bleeding in the right lung lobes, which was managed successfully with interventional radiology.

**Table 1:** Hemoptysis in SVC syndrome is associated with mortality.

Year	Clinical Features	Mortality	Reference
2009	A 75-year-old male presented with marked swelling of his face and arms and shortness of breath complicated by hematemesis and hemoptysis.	No; symptoms improved with the removal of the right internal jugular hemodialysis catheter.	Gopaluni S et al. [12]
2014	A 38-year-old male with hypertension and end-stage renal disease (ESRD) on HD presented with intermittent episodes of hemoptysis.	Yes, from cardiac arrest.	Arnous N et al. [13]
2019	A 23-year-old female with ESRD on chronic HD presented with hematemesis and hemoptysis.	No; symptoms improved with the restoration of SVC patency.	Tannu M et al. [8]
2023	A 27-year-old man known to have end-stage kidney disease (ESKD) on hemodialysis presented with shortness of breath and life-threatening hemoptysis.	Yes, from cardiac arrest.	Al Saadi W et al. [14]

Patients with SVC can commonly present with pleural effusion, as in our case. Effusions in such settings are mostly believed to be transudative in nature, caused by an increase in hydrostatic pressure, like those seen with congestive heart failure or nephrosis [9]. However, Todd et al. reported cases of non-malignant SVC syndrome patients with exudative pleural effusions, likely due to multifactorial causes [10,11].

A CT scan of the chest with intravenous contrast is required to confirm the diagnosis of SVC syndrome. Management of SVC syndrome with thrombus typically involves anticoagulation and stent placement for recanalization [14,15]. Unfortunately, no guidelines outline the duration and follow-up for anticoagulation, which makes it more challenging in patients with GI bleeding or hematemesis associated with obstruction [16]. Revascularization of SVC with a stent shows overall 75% immediate relief of symptoms [17]. In the analysis of 136 patients with endovascular treatment and 87 patients with surgical management of benign causes of SVC syndrome, the endovascular approach had a 95.9% success rate compared to the 100% success rate of open approaches. Neither approach noted 30-day mortality [18]. However, in cases where these are not viable options, alternative strategies like thoracentesis, pleurodesis, or pleuroperitoneal shunt placement may be considered for SVC syndrome-associated complications [9,19,20].

## Conclusions

Hemoptysis is one of the least recognized manifestations of SVC syndrome and has been very rarely mentioned in the literature. This necessitates considering SVC syndrome as a differential diagnosis when a patient presents with hemoptysis in the right clinical context. This case report also highlights dilemmas as well as strategies for managing SVC syndrome in Jehovah's Witnesses patients who refuse transfusions due to religious beliefs. Managing such a case requires a fine balance between the patient's autonomy and critical care demands.
